# Supplementary material for: An oncopeptide regulates m6A recognition by the m6A reader IGF2BP1 and tumorigenesis
Source: Nat Commun. 2020 Apr 3;11:1685. doi: 10.1038/s41467-020-15403-9 (PMC7125119; doi:10.1038/s41467-020-15403-9)
Supplement: Supplementary file 1 — Supplementary Information [file 41467_2020_15403_MOESM1_ESM.pdf]

Supplementary information

An oncopeptide regulates m^6A recognition by the m^6A reader IGF2BP1 and tumorigenesis

Zhu *et al*.

Supplementary Figures

```
TGCACACATCTTCTTCTCCAAGGTTTGTGTGCAGAACATCCTGCCCATGCTGACCCAGGAGCTTCAGT
TGGCACCTGCCCCAGTCCAGCCTCTGGGAACCATGCAGCAGCTCCCAGCGGCCCTGCACCCACCAC
CAGCATCCGTTTCACCTGCAGTTGAAGATCCGTGAGGTGCCCAGAAGATCATGCAGTCATCAGTCCC
ACGGAGCAGCCCGCGAGGCTGAGGCTCCTCCCACTGGACCGCCCCCCAACTGGCACCACTGCTGC
CCCTGCCCCTACTCTCAGCCTCACGTGACTCTCGGGCAGAGGCAGTGGTGGGGCAGCCAGGGCAG
CGTCAAGAGTCTGAGCCAGCTGCAGGACAAATTCGAGCATCTTAAAATGATTCAACAGGAGGAGATA
                                              M  I  Q  Q  E  E  I
AGGAAGCTCGAGGAAGAGAAAAAACAACTGGAAGGAGAAATCATAGATTTTTATAAAATGAAAGCTGC
 R  K  L  E  E  E  K  K  Q  L  E  G  E  I  I  D  F  Y  K  M  K  A  A
CTCTGAAGCACTGCAGACTCAGCTGAGCACTGATACAAAGAAAGACAAACATCCTGATCCATATGAAT
  S  E  A  L  Q  T  Q  L  S  T  D  T  K  K  D  K  H  P  D  P  Y  E
TCCTCTTATTAAGAAAAATAAAGCATCCAGGATTCAATGAAGAACTATCACCTTGTTAATCATTCAGAA
F  L  L  L  R  K  I  K  H  P  G  F  N  E  E  L  S  P  C
ACATATTGCAGACTTAAGCCATTTTTGATACAGATACTGAAACAATTACTTTCTAAGAGCAAACTTGAA
GGTATGGATAAGGCCCTGAGTCATCTTCCTGAGCTGAATGATAGTTAAGCT
```

Supplementary Figure 1. The amino acid sequences of the RBRP peptide are encoded by the *LINC00266-1* ORF in *H. sapiens*. The *LINC00266-1*-coding ORF sequences are indicated in blue. The ATG start codon and TAA stop codon are indicated in green and underlined, and the amino acid sequences of the RBRP peptide are indicated in pink.

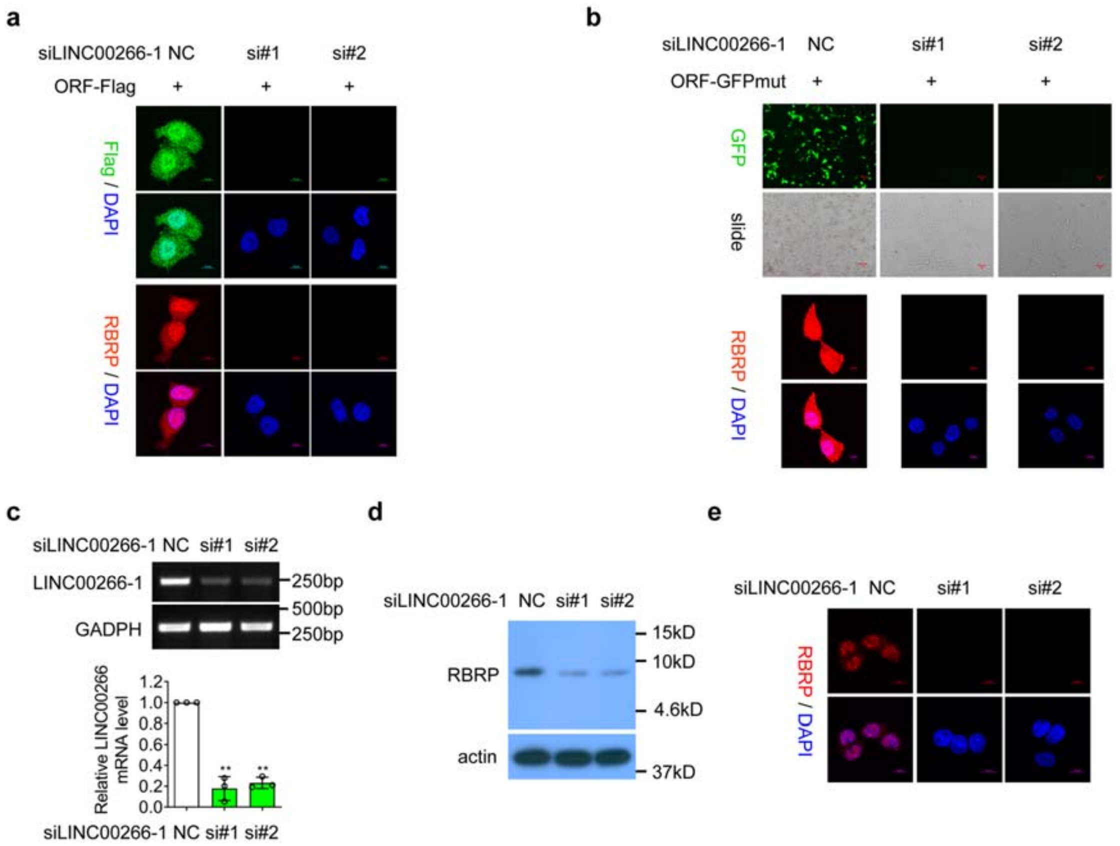

Supplementary Figure 2. An anti-RBRP antibody specifically detects the RBRP peptide. **a** HCT-116 cells were cotransfected with *LINC00266-1* ORF-Flag constructs together with anti-*LINC00266-1* siRNAs; RBRP-Flag fusion peptide was detected by immunostaining with anti-Flag and anti-RBRP antibodies. Scale bar: 10 µm. **b** HCT-116 cells were cotransfected with *LINC00266-1* ORF-GFPmut constructs together with anti-*LINC00266-1* siRNAs; RBRP-GFP fusion peptide was directly visualized by fluorescence or detected by immunostaining with anti-RBRP antibodies. Scale bar: 100 µm. **c** HCT-116 cells were transfected with anti-*LINC00266-1* siRNAs, and the *LINC00266-1* lncRNA levels were determined by RT-PCR (upper panel) and qRT-PCR (lower panel) (n=3, three independent experiments for qRT-PCR). **d, e** HCT-116 cells were transfected with anti-*LINC00266-1* siRNAs, and RBRP levels were detected by Western blotting (**d**) and immunostaining (**e**) with an anti-RBRP antibody. Scale bar: 10 µm. Two-tailed unpaired Student's *t*-test. The data are represented as the means ± SD. **p<0.01. Source data are provided as a Source Data file.

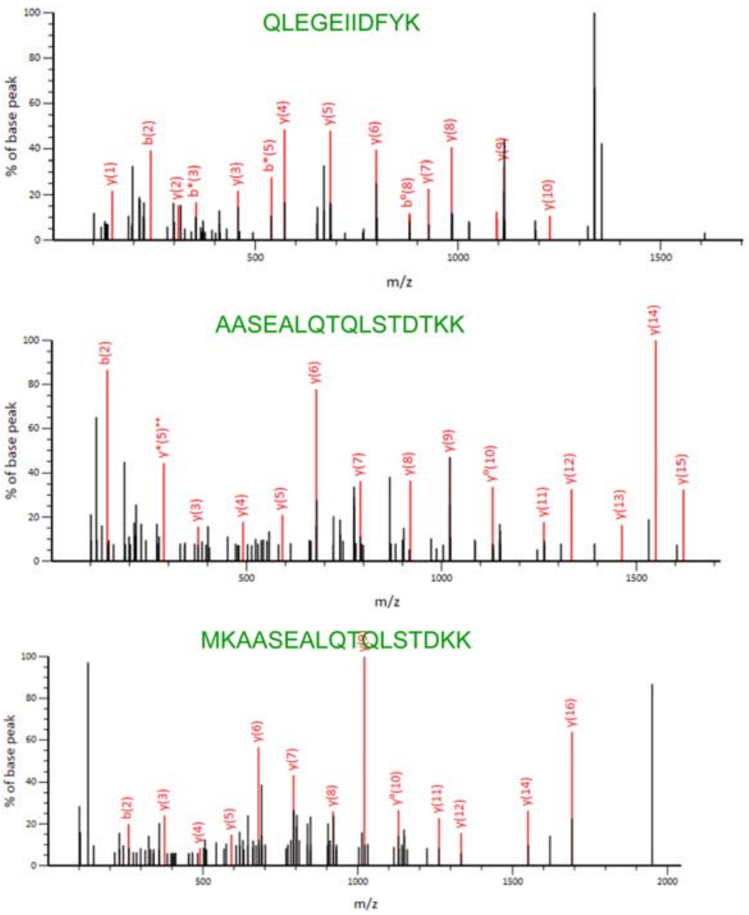

Supplementary Figure 3. RBRP peptide was identified as endogenously, naturally expressed in cancer tissues by a mass spectrometry assay. Three unique peptide fragments in the endogenously, naturally expressed RBRP peptide in cancer tissues were identified using mass spectrometry. Representative MS/MS spectra of three identified peptide fragments in the endogenously, naturally expressed RBRP peptide.

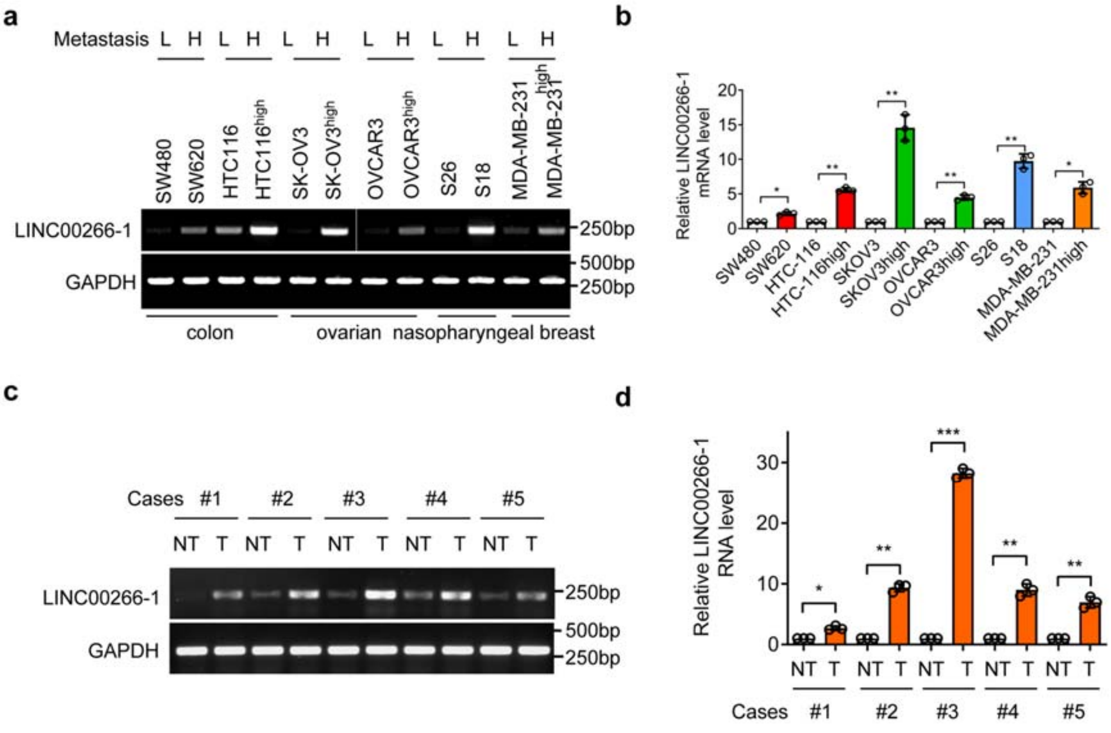

Supplementary Figure 4. *LINC00266-1* lncRNA levels are upregulated in highly metastatic cancer cell sublines and primary colon cancer tissues compared with the levels in their parental cell lines and corresponding adjacent NT, respectively. **a, b** *LINC00266-1* lncRNA levels were determined in the indicated cancer cell lines and sublines with different metastatic properties by RT-PCR (**a**) and qRT-PCR (**b**) (n=3, three independent experiments for qRT-PCR). **c, d** *LINC00266-1* lncRNA levels were analyzed in five pairs of matched fresh primary CRC tissues (T) and their corresponding adjacent NT by RT-PCR (**c**) and qRT-PCR (**d**) (n=3, three independent experiments for qRT-PCR). Two-tailed unpaired Student's *t*-test. The data are represented as the means ± SD. *p<0.05, **p<0.01 or ***p<0.001, ns indicates no significance. Source data are provided as a Source Data file.

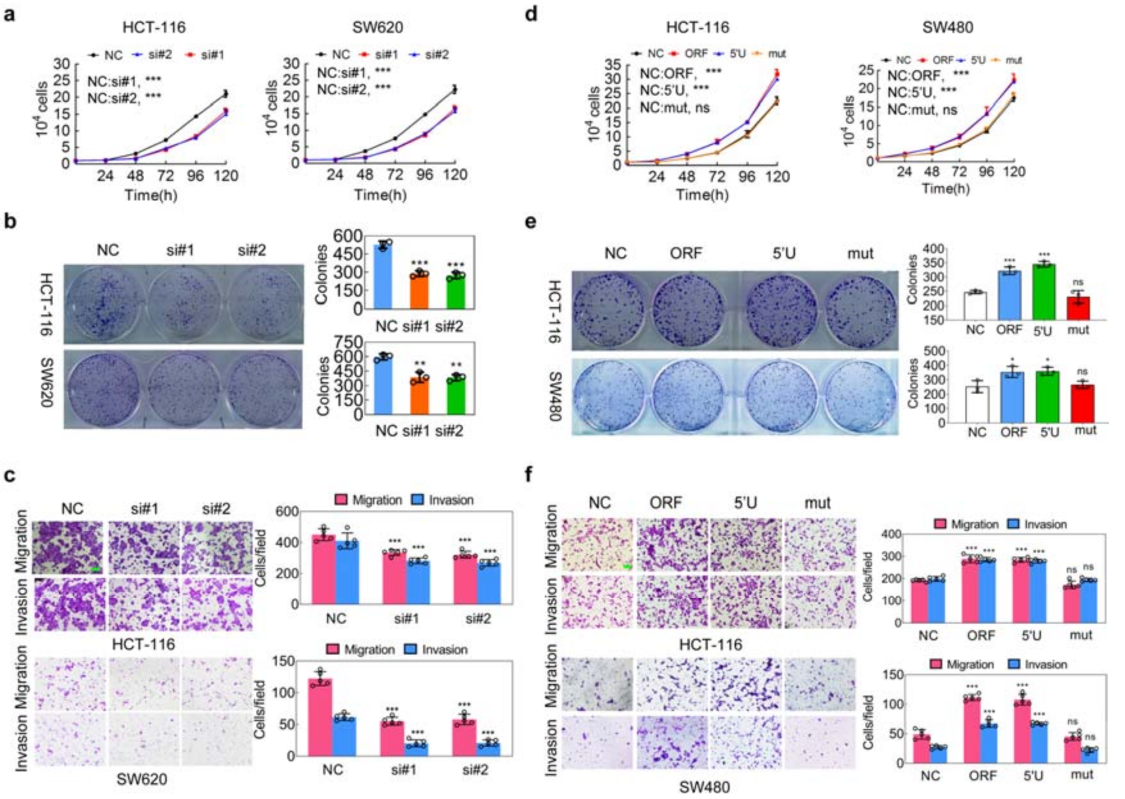

Supplementary Figure 5. The RBRP peptide, not its *LINC00266-1* lncRNA, promotes CRC cell growth, colony formation, migration and invasion, while *LINC00266-1* knockdown exhibited the opposite effects. **a-c** CRC cells HCT-116 and SW620 were transfected with two anti-*LINC00266-1* siRNAs, and cell proliferation (**a**) (n=3 independent experiments), colony formation (**b**) (n=3 independent experiments), migration and invasion (**c**) (n=5 independent experiments) were determined. **d-f** The indicated CRC cells were transfected with the indicated *LINC00266-1* constructs, and cell proliferation (**d**) (n=3 independent experiments), colony formation (**e**) (n=3 independent experiments), migration and invasion (**f**) (n=5 independent experiments) were determined. Scale bar: 50 μm. Two-tailed unpaired Student's *t*-test unless specifically stated, two-way ANOVA in (a) and (d). The data are represented as the means ± SD. *p<0.05, **p<0.01 or ***p<0.001, ns indicates no significance. Source data are provided as a Source Data file.

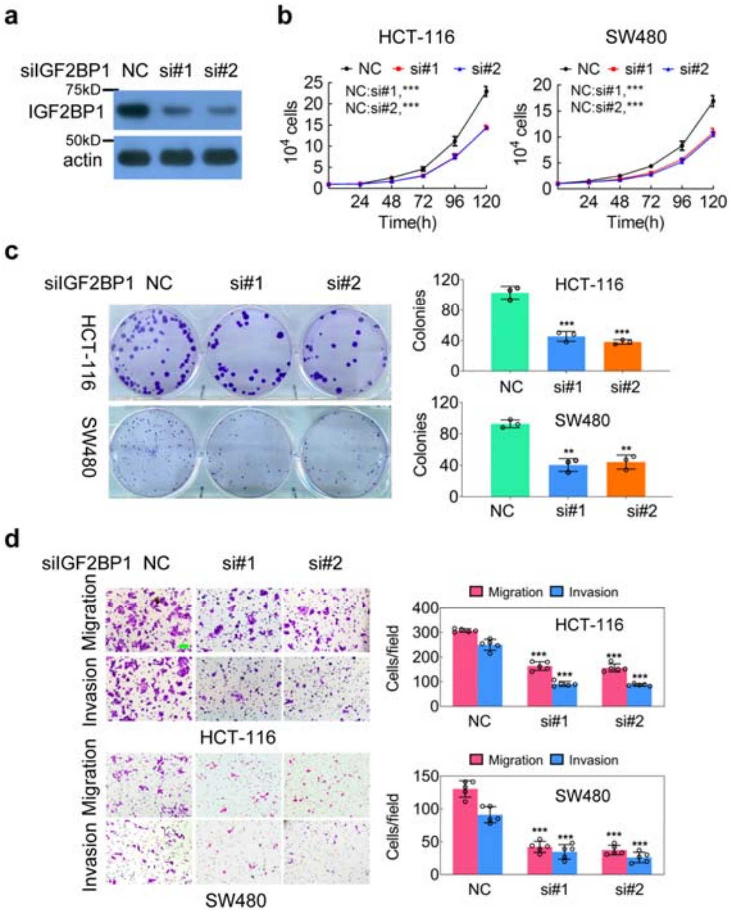

Supplementary Figure 6. Knockdown of *IGF2BP1* inhibits CRC cell proliferation, colony formation, migration and invasion, phenocopying the functions of RBRP knockdown. CRC cells HCT-116 and SW480 were transfected with two anti-*IGF2BP1* siRNAs, and the IGF2BP1 protein level (**a**), cell proliferation (**b**) (n=3 independent experiments), colony formation (**c**) (n=3 independent experiments), migration and invasion (**d**) (n=5 independent experiments) were determined. Scale bar: 50 μm. Two-tailed unpaired Student's *t*-test unless specifically stated, two-way ANOVA in (b). The data are represented as the means ± SD. *p<0.05, **p<0.01 or ***p<0.001, ns indicates no significance. Source data are provided as a Source Data file.

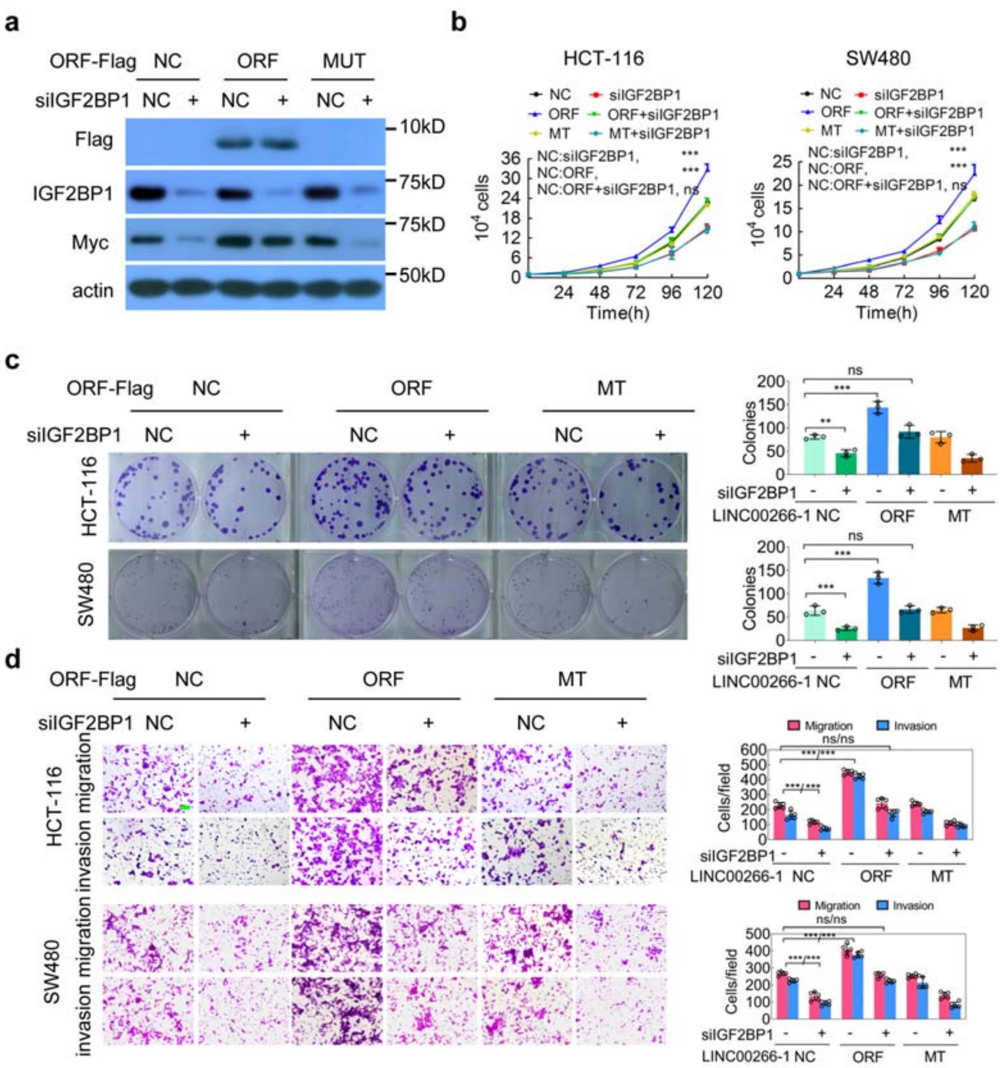

Supplementary Figure 7. Knockdown of *IGF2BP1* blocked the enhancement of cancer phenotypes induced by RBRP overexpression. CRC cells HCT-116 and SW480 were cotransfected with the indicated *LINC00266-1* vectors and anti-*IGF2BP1* siRNAs, and the indicated protein levels (**a**), cell proliferation (**b**) (n=3 independent experiments), colony formation (**c**) (n=3 independent experiments), migration and invasion (**d**) (n=5 independent experiments) were determined. Scale bar: 50 μm. Two-tailed unpaired Student's *t*-test unless specifically stated, two-way ANOVA in (b). The data are represented as the means ± SD. **p<0.01 or ***p<0.001, ns indicates no significance. Source data are provided as a Source Data file.

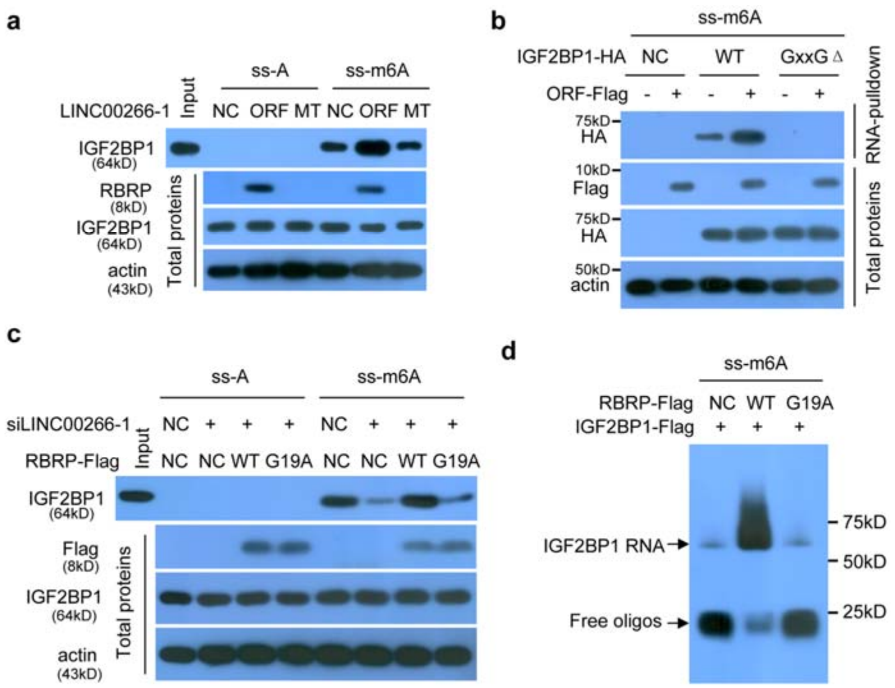

Supplementary Figure 8. RBRP increases the m^{6}A recognition by the m^{6}A reader IGF2BP1 on RNAs. **a** The *in vitro* binding of IGF2BP1 to m^{6}A-unmethylated (ss-A) or methylated (ss-m^{6}A) single-stranded RNA probes was investigated in HCT-116 cells stably expressing LINC00266-1 ORF-Flag or 5'UTR-ORFmut-Flag (MT) by RNA pull-down assays. **b** The WT or GxxG-mutated (GxxGΔ) *IGF2BP1-HA* plasmids together with *LINC00266-1* ORF-Flag vectors were cotransfected into cells, and the *in vitro* binding of IGF2BP1 on ss-m^{6}A RNA probes was investigated by RNA pull-down assays. **c** WT or G19A-mutated *RBRP-Flag* vectors were transfected into HCT-116 cells with the stable knockdown of *LINC00266-1* expression, and the *in vitro* binding of IGF2BP1 on ss-A or ss-m^{6}A RNA probes was analyzed by an RNA pull-down assay. **d** Recombinant IGF2BP1 protein, WT or G19A-mutated RBRP peptide and ss-m^{6}A RNA probes were incubated, and the binding capability of IGF2BP1 on ss-m^{6}A RNA probes was analyzed by an RNA EMSA assay. Source data are provided as a Source Data file.

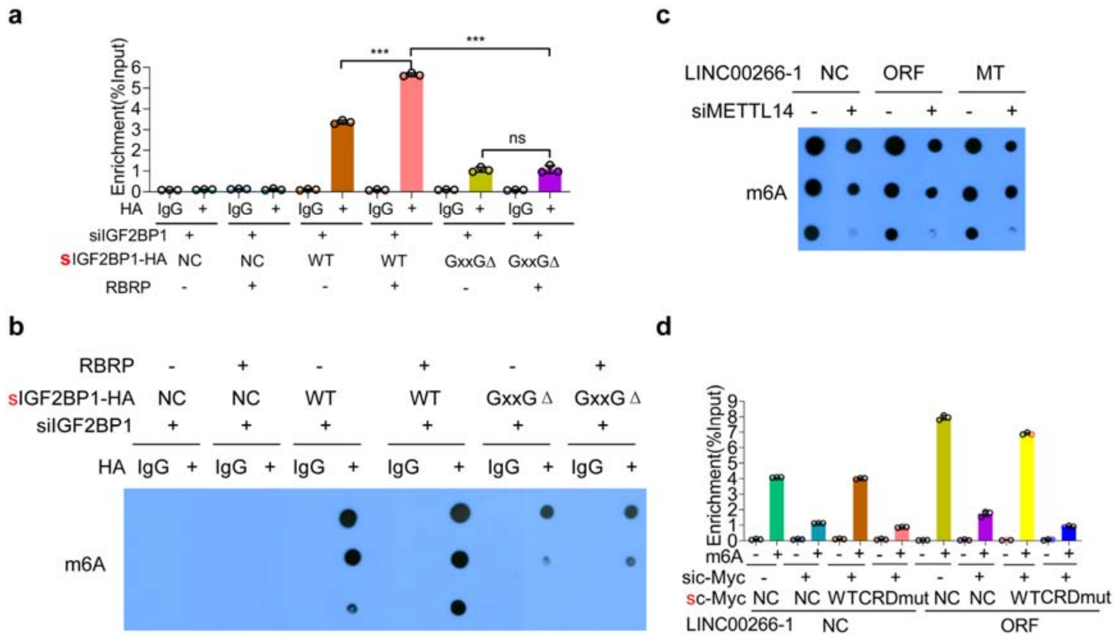

Supplementary Figure 9. RBRP increases the recognition of the m^{6}A reader IGF2BP1 for m^{6}A-modified *c-Myc* CRD mRNA. **a** The WT or GxxG-mutated *sIGF2BP1-HA* plasmids, which are resistant to anti-*IGF2BP1* siRNA, were cotransfected with both anti-*IGF2BP1* siRNA and the *LINC00266-1* ORF vector, into HEK293T cells; the *in vivo* binding of IGF2BP1 to *c-Myc* CRD mRNA was determined by an RIP-qPCR assay (n=3 independent experiments). **b** The m^{6}A levels in IGF2BP1-bound RNAs were determined in (**a**). **c** The m^{6}A levels in IGF2BP1-bound RNAs were reduced by silencing the m^{6}A writer *METTL14*. **d** The m^{6}A levels in *c-Myc* CRD mRNA were determined in WT or A-to-U-mutated *c-Myc* CRD mRNA by a gene-specific m^{6}A assay (n=3 independent experiments). The cells were treated as in Figure 5i. Two-tailed unpaired Student's *t*-test. The data are represented as the means ± SD. ***p<0.001, ns indicates no significance. Source data are provided as a Source Data file.

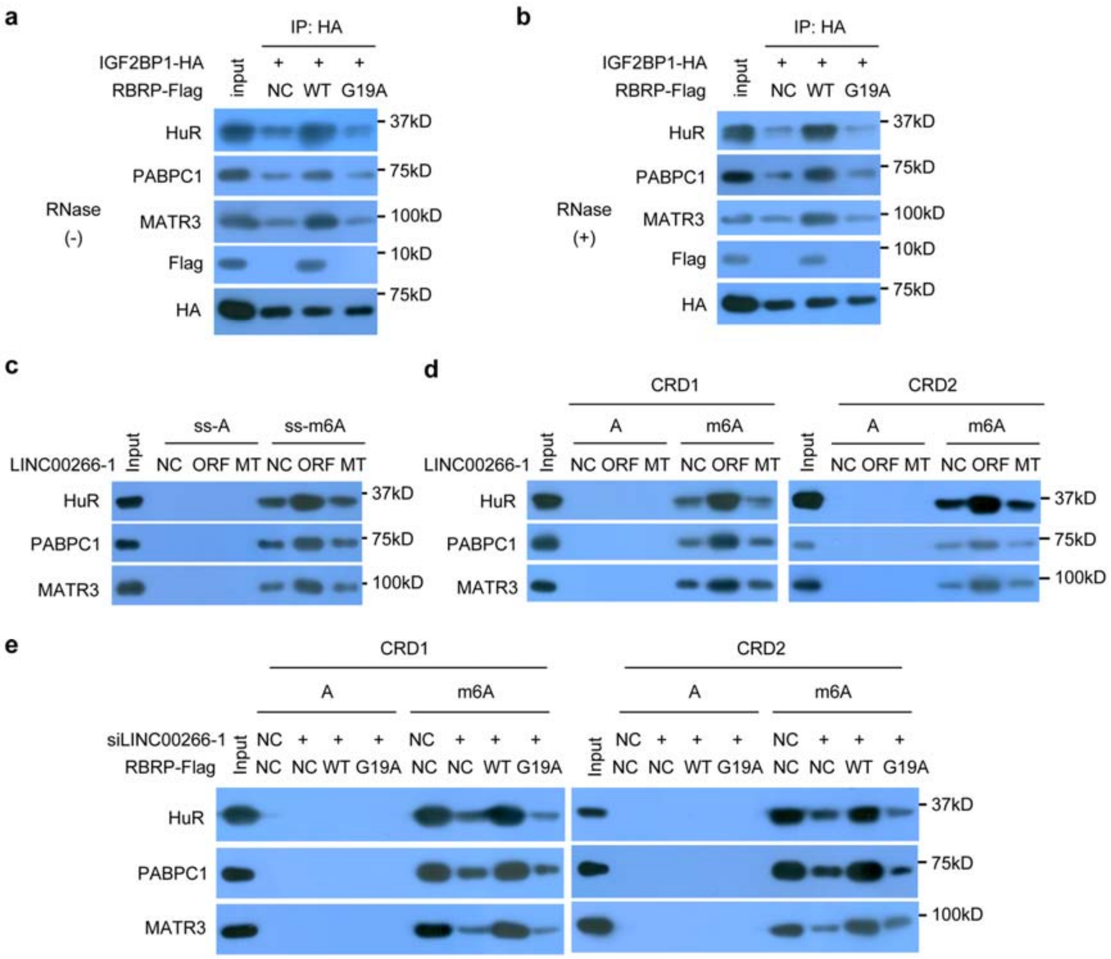

Supplementary Figure 10. RBRP increases the binding of the RNA stabilizers HuR, PABPC1 and MATR3 to IGF2BP1 and m^{6}A RNA. **a, b** The interactions of IGF2BP1 with the RNA stabilizers HuR, PABPC1 and MATR3 were investigated in the absence (**a**) or presence (**b**) of RNase A treatment, when the WT or G19A-mutated RBRP-Flag was overexpressed. **c** The *in vitro* binding of these RNA stabilizers to ss-A or ss-m^{6}A RNA probes was investigated by RNA pull-down assays when *LINC00266-1* ORF-Flag or 5'UTR-ORFmut-Flag was overexpressed in HCT-116 cells. **d** The *in vitro* binding of these RNA stabilizers on m^{6}A-unmethylated or methylated *c-Myc* CRD mRNA oligos was investigated by RNA pull-down assays when *LINC00266-1* ORF-Flag or 5'UTR-ORFmut-Flag was overexpressed in HCT-116 cells. **e** The *in vitro* binding of these RNA stabilizers on m^{6}A-unmethylated or methylated *c-Myc* CRD mRNA oligos was investigated by RNA pull-down assays when the WT or G19A-mutated RBRP-Flag was overexpressed in HCT-116 cells stably silencing *LINC00266-1* expression. Source data are provided as a Source Data file.

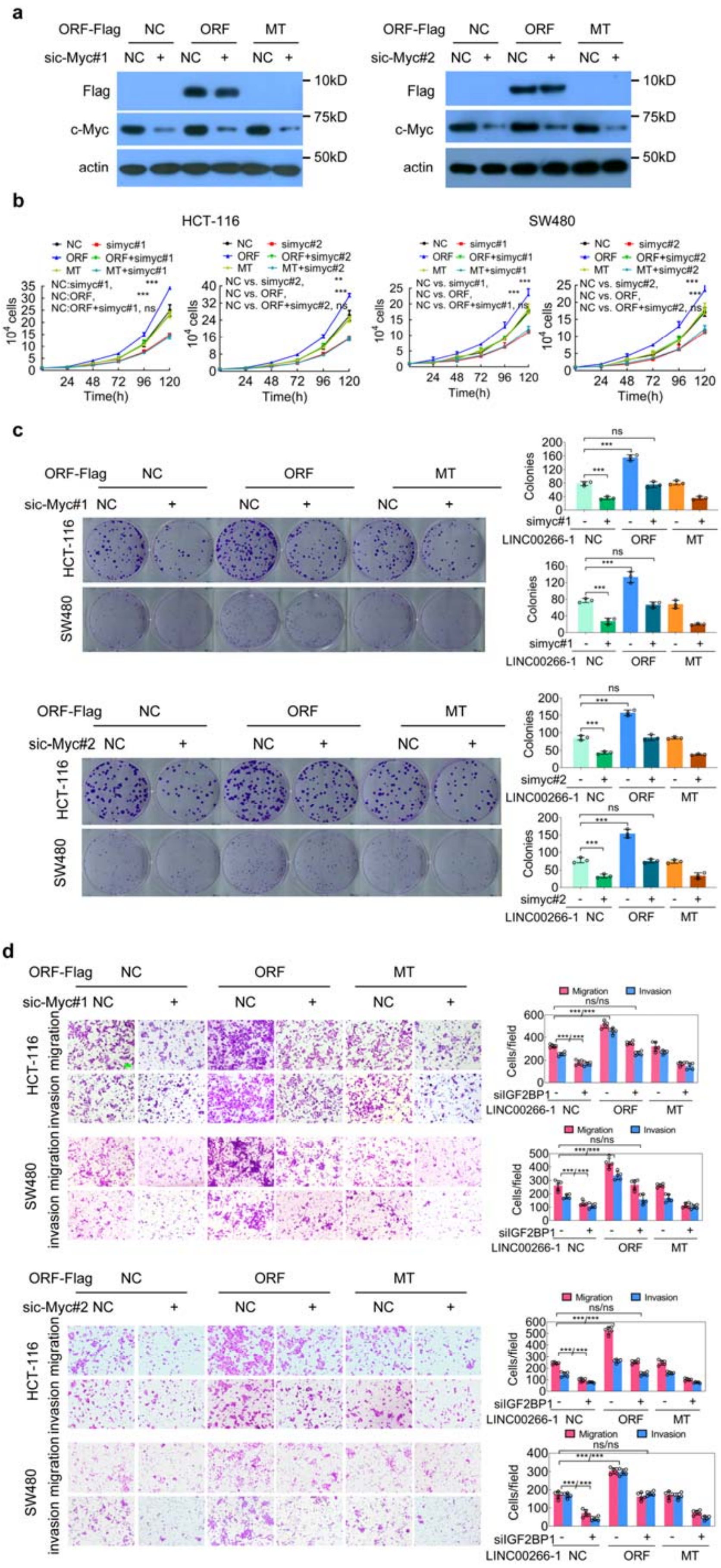
a
ORF-Flag NC ORF MT
sic-Myc#1 NC + NC + NC +
Flag
c-Myc
actin
10kD
75kD
50kD
sic-Myc#2 NC + NC + NC +
b
HCT-116
SW480
NC simyc#1 ORF ORF+simyc#1 MT MT+simyc#1
NC simyc#2 ORF ORF+simyc#2 MT MT+simyc#2
NC:simyc#1, NC:ORF, NC:ORF+simyc#1, ns
NC vs. simyc#2, NC vs. ORF, NC vs. ORF+simyc#2, ns
NC vs. simyc#1, NC vs. ORF, NC vs. ORF+simyc#1, ns
10⁴ cells
Time(h)
c
ORF-Flag NC ORF MT
sic-Myc#1 NC + NC + NC +
sic-Myc#2 NC + NC + NC +
HCT-116
SW480
Colonies
simyc#1
simyc#2
LINC00266-1 NC ORF MT
d
ORF-Flag NC ORF MT
sic-Myc#1 NC + NC + NC +
sic-Myc#2 NC + NC + NC +
HCT-116 migration invasion
SW480 migration invasion
Migration Invasion
Cells/field
siIGF2BP1
LINC00266-1 NC ORF MT

Supplementary Figure 11. RBRP exerts its oncogenic functions through c-Myc. CRC cells HCT-116 and SW480 were cotransfected with the indicated *LINC00266-1* vectors and anti-*c-Myc* siRNAs, and the indicated protein levels (**a**), cell proliferation (**b**) (n=3 independent experiments), colony formation (**c**) (n=3 independent experiments), migration and invasion (**d**) (n=5 independent experiments) were determined. Scale bar: 50 μm. Two-tailed unpaired Student's *t*-test unless specifically stated, two-way ANOVA in (b). The data are represented as the means ± SD. *$p<0.05$, **$p<0.01$ or ***$p<0.001$, ns indicates no significance. Source data are provided as a Source Data file.

Supplementary Tables

Supplementary Table 1. Correlations between RBRP oncopeptide levels and clinicopathological features in 90 colon cancer cases. Statistical analysis was performed using Pearson Chi-square test.

clinical features	All cases	RBRP oncopeptide		*p* value*
		Low	High	
Sex				0.561
Female	43	20	23	
Male	47	19	28	
Age(Years)				0.068
＜60	29	17	12	
≥60	61	22	39	
Histological Grade				0.824
G1-G2	77	33	44	
G3-G4	13	6	7	
Tumor size				0.333
＜5cm	36	18	18	
≥5cm	53	21	32	
pT status				0.522
1-2	11	6	5	
3-4	79	33	46	
pN status				0.065
0-1	82	38	44	
2	8	1	7	
Clinical Stage				0.027
Ⅰ-ⅡA	48	26	22	
ⅡB-Ⅳ	42	13	29	

*Pearson Chi-square test.

Supplementary Table 2. Univariate and multivariate analysis of different prognostic parameters in 90 colon cancer patients. Statistical analysis was performed using Cox proportional hazard model.

Clinical featutures	OS(months)			
	Univariate analysis		Multivariatie analysis	
	HR(95% CI)	*p* value*	HR(95% CI)	*p* value*
Age (≥60y vs.<60y)	0.90(0.45-1.81)	0.763	0.70(0.34-1.47)	0.348
Sex (Male vs. Female)	0.80(0.41-1.57)	0.516	0.78(0.38-1.62)	0.507
Tumor size (≥5cm vs.<5cm)	1.79(0.88-3.66)	0.111	1.58(0.71-3.56)	0.265
pT status (3-4 vs.1-2)	2.61(0.63-10.88)	0.188	1.12(0.24-5.37)	0.884
pN status (2 vs. 0-1)	4.22(1.83-9.76)	0.001	2.47(0.98-6.23)	0.055
Histological Grade (G3-G4 vs. G1-G2)	3.54(1.65-7.63)	0.001	3.77(1.63-8.76)	0.002
RBRP oncopeptide (scores 4-7 vs. 0-3)	7.92(2.76-22.49)	0.000	8.26(2.84-24.02)	0.000

*Cox proportional hazard model.

Supplementary Table 3. The sequences of genes, siRNAs, oligos, m6A-oligos and the RT-PCR/qRT-PCR primers used in this study.

Name	Sequences
LINC00266-1<br>ORF-Flag	*ATG*ATTCAACAGGAGGAGATAAGGAAGCTCGAGGA<br>AGAGAAAAAACAACTGGAAGGAGAAATCATAGATT<br>TTTATAAAATGAAAGCTGCCTCTGAAGCACTGCAGA<br>CTCAGCTGAGCACTGATACAAAGAAAGACAAACAT<br>CCTGATCCATATGAATTCCTCTTATTAAGAAAAATAA<br>AGCATCCAGGATTCAATGAAGAACTATCACCTTGTG<br>ACTACAAGGACGACGATGACAAG*TAA*
LINC00266-1<br>5'UTR-ORF-Flag	TGCACACATCTTCTTCTCCAAGGTTTGTGTGCAGAA<br>CATCCTGCCCATGCTGACCCAGGAGCTTCAGTTGGC<br>ACCTGCCCCAGTCCAGCCTCTGGGAACCATGCAGC<br>AGCTCCCAGCGGCCCTGCACCCACCACCAGCATCC<br>GTTTCACCTGCAGTTGAAGATCCGTGAGGTGCCCAG<br>AAGATCATGCAGTCATCAGTCCCACGGAGCAGCCC<br>GCGAGGCTGAGGCTCCTCCCACTGGACCGCCCCCC<br>AACTGGCACCACTGCTGCCCCTGCCCCTACTCTCAG<br>CCTCACGTGACTCTCGGGCAGAGGCAGTGGTGGGG<br>CAGCCAGGGCAGCGTCAAGAGTCTGAGCCAGCTGC<br>AGGACAAATTCGAGCATCTTAAA*ATG*ATTCAACAGG<br>AGGAGATAAGGAAGCTCGAGGAAGAGAAAAAACA<br>ACTGGAAGGAGAAATCATAGATTTTTATAAAATGAA<br>AGCTGCCTCTGAAGCACTGCAGACTCAGCTGAGCA<br>CTGATACAAAGAAAGACAAACATCCTGATCCATATG<br>AATTCCTCTTATTAAGAAAAATAAAGCATCCAGGATT<br>CAATGAAGAACTATCACCTTGTGACTACAAGGACGA<br>CGATGACAAG*TAA*
LINC00266-1<br>5'UTR-ORFmut-Flag	TGCACACATCTTCTTCTCCAAGGTTTGTGTGCAGAA<br>CATCCTGCCCATGCTGACCCAGGAGCTTCAGTTGGC<br>ACCTGCCCCAGTCCAGCCTCTGGGAACCATGCAGC<br>AGCTCCCAGCGGCCCTGCACCCACCACCAGCATCC<br>GTTTCACCTGCAGTTGAAGATCCGTGAGGTGCCCAG<br>AAGATCATGCAGTCATCAGTCCCACGGAGCAGCCC<br>GCGAGGCTGAGGCTCCTCCCACTGGACCGCCCCCC<br>AACTGGCACCACTGCTGCCCCTGCCCCTACTCTCAG<br>CCTCACGTGACTCTCGGGCAGAGGCAGTGGTGGGG<br>CAGCCAGGGCAGCGTCAAGAGTCTGAGCCAGCTGC<br>AGGACAAATTCGAGCATCTTAAA*ATT*ATTCAACAGG<br>AGGAGATAAGGAAGCTCGAGGAAGAGAAAAAACA<br>ACTGGAAGGAGAAATCATAGATTTTTATAAAATGAA

	AGCTGCCTCTGAAGCACTGCAGACTCAGCTGAGCA<br>CTGATACAAAGAAAGACAAACATCCTGATCCATATG<br>AATTCCTCTTATTAAGAAAAATAAAGCATCCAGGATT<br>CAATGAAGAACTATCACCTTGTGACTACAAGGACGA<br>CGATGACAAG*TAA*
siLINC00266-1#1:	sense: 5’-CGUUUCACCUGCAGUUGAATT-3’<br>antisense: 5’-UUCAACUGCAGGUGAAACGTT-3’
siLINC00266-1#2:	sense: 5’-CAUUCAGAAACAUAUUGCATT-3’<br>antisense: 5’-UGCAAUAUGUUUCUGAAUGTT-3’
LINC00266-1 shRNA (target to its 3’UTR)	CAUUCAGAAACAUAUUGCA
siIGF2BP1#1:	sense: 5’-CUCCGGGAAAGUAGAAUUATT-3’<br>antisense: 5’-UAAUUCUACUUUCCCGGAGTT-3’
siIGF2BP1#2:	sense: 5’-GGCCCAUAAUAACUUUGUATT-3’<br>antisense: 5’-UACAAAGUUAUUAUGGGCCTT-3’
siMETTL14#1:	sense: 5’-GGAUGAAGGAGAGACAGAUTT-3’<br>antisense: 5’-AUCUGUCUCUCCUUCAUCCTT-3’
siMETTL14#2:	sense: 5’-GCAGCACCUCGAUCAUUUATT-3’<br>antisense: 5’-UAAAUGAUCGAGGUGCUGCTT-3’
sic-Myc#1:	sense: 5’-GUGCAGCCGUAUUUCUACUTT-3’<br>antisense: 5’-AGUAGAAAUACGGCUGCACTT-3’
sic-Myc#2:	sense: 5’-GAACACACAACGUCUUGGATT-3’<br>antisense: 5’-UCCAAGACGUUGUGUGUUCTT-3’
NC siRNA:	sense: 5’-GCACAAGCUGGAGUACAACUACATT-3’<br>antisense:5’-UGUAGUUGUACUCCAGCUUGUGCTT-3’
ss-A:	Biotin-CGUCUCGGACUCGGACUGCU
ss-m6A:	Biotin-CGUCUCGGA*(m6A)CUCGGA*(m6A)CUGCU
c-Myc CRD1-A:	Biotin-GAAGAGGACUUGUUGCGGAAACGACGAGAA<br>CAGUU

c-Myc CRD1-m6A:	Biotin-GAAGAGGA*(m6A)CUUGUUGCGGAAACGAC GAGAA*(m6A)CAGUU
c-Myc CRD2-A:	Biotin-AGUUGAAACACAAACUUGAACAGCUACGGA ACUCUU
c-Myc CRD2-m6A:	Biotin-AGUUGAAA*(m6A)CACAAA*(m6A)CUUGAA*(m6A)CAGCUACGGAA*(m6A)CUCUU
RT-PCR	
LINC00266-1	Forward: TGCCCAGAAGATCATGCAGTC<br>Reverse: TCCTCGAGCTTCCTTATCTCC
IGF2BP1	Forward: ACATCGGCAACCTCAACGAGA<br>Reverse: GACATTCACCACTGCCGTCT
c-Myc	Forward: CTCGGTGCAGCCGTATTT<br>Reverse: CGGGTCGCAGATGAAACT
METTL14	Forward: ACTGCCTCATGGGGATCAAA<br>Reverse: GCCAGCCTGGTCGAATTGTA
GAPDH	Forward: CGGAGTCAACGGATTTGGTCGTAT<br>Reverse: AGCCTTCTCCATGGTGGTGAAGAC
qRT-PCR	
LINC00266-1	Forward: TGCCCAGAAGATCATGCAGTC<br>Reverse: TCCTCGAGCTTCCTTATCTCC
c-Myc CRD	Forward: AACACACAACGTCTTGGAG<br>Reverse: TTACGCACAAGAGTTCCGTAG
GAPDH	Forward: GAAGGTGAAGGTCGGAGTC<br>Reverse: AAGATGGTGATGGGATTTC
