# Supplementary material for: An oncopeptide regulates m6A recognition by the m6A reader IGF2BP1 and tumorigenesis
Source: Nat Commun. 2020 Apr 3;11:1685. doi: 10.1038/s41467-020-15403-9 (PMC7125119; doi:10.1038/s41467-020-15403-9)
Supplement: Supplementary file 3 — Description of Additional Supplementary Files [file 41467_2020_15403_MOESM3_ESM.pdf]

Description of Additional Supplementary Files

File Name: Supplementary Data 1
Description: The differentially expressed ribosome-bound lncRNAs between SW620 and SW480.

File Name: Supplementary Data 2
Description: The proteins interacted with RBRP were identified by Co-IP together with proteomics.
